# Hypoxia/reperfusion predisposes to atherosclerosis

**DOI:** 10.1371/journal.pone.0205067

**Published:** 2018-10-05

**Authors:** Richard Finsterwalder, Minu Karthika Ganesan, Heide Leb, Andreas Habertheuer, José Basílio, Irene Lang, Milica Krunic, Dominik Wiedemann, Peter Petzelbauer

**Affiliations:** 1 Skin and Endothelium Research Division (SERD), Department of Dermatology, Medical University of Vienna, Vienna, Austria; 2 Division of Cardiovascular Surgery, Hospital of the University of Pennsylvania, Perelman School of Medicine, Philadelphia, Pennsylvania, United States of America; 3 Department for Vascular Biology and Thrombosis Research, Medical University of Vienna, Vienna, Austria; 4 Department of Internal Medicine II, Division of Cardiology, Vienna General Hospital, Medical University of Vienna, Vienna, Austria; 5 Center for Integrative Bioinformatics Vienna, Max. F. Perutz Laboratories, University of Vienna, Vienna, Medical University of Vienna, Vienna, Austria; 6 Department of Cardiac Surgery, Medical University of Vienna, Vienna, Austria; Qatar University College of Health Sciences, QATAR

## Abstract

Surgical interventions on blood vessels bear a risk for intimal hyperplasia and atherosclerosis as a consequence of injury. A specific feature of intimal hyperplasia is the loss of vascular smooth muscle cell (VSMC) differentiation gene expression. We hypothesized that immediate responses following injury induce vascular remodeling. To differentiate injury due to trauma, reperfusion and pressure changes we analyzed vascular responses to carotid artery bypass grafting in mice compared to transient ligation. As a control, the carotid artery was surgically laid open only. In both, bypass or ligation models, the inflammatory responses were transient, peaking after 6h, whereas the loss of VSMC differentiation gene expression persisted. Extended time kinetics showed that transient carotid artery ligation was sufficient to induce a persistent VSMC phenotype change throughout 28 days. Transient arterial ligation in ApoE knockout mice resulted in atherosclerosis in the transiently ligated vascular segment but not on the not-ligated contralateral side. The VSMC phenotype change could not be prevented by anti-TNF antibodies, Sorafenib, Cytosporone B or N-acetylcysteine treatment. Surgical interventions involving hypoxia/reperfusion are sufficient to induce VSMC phenotype changes and vascular remodeling. In situations of a perturbed lipid metabolism this bears the risk to precipitate atherosclerosis.

## Introduction

Arteries and veins consist of 3 layers; the adventitia, largely constituted of connective tissue and fibroblasts, the media mainly containing vascular smooth muscle cells (VSMCs) and the intima. Separated from the media by the internal elastic lamina, the intima consists of loose connective tissue intermingled with few VSMCs and a monolayer of endothelial cells (ECs) resting on a basal membrane forming the interface to the bloodstream. VSMCs of the intima are known to respond to almost any sort of physical or chemical injury by proliferation and migration, resulting in thickening which is termed intimal hyperplasia (IH).[1]. Of note, distinctions should be made between acute versus chronic injury, the latter occurring with aging; age-associated vascular remodeling is a risk factor for atherosclerosis in humans.[2–4] Excessive IH can cause morbidity by narrowing the vessel lumen or by priming the vessel for atherosclerosis.[5] This is of special importance for patients subjected to coronary revascularization procedures, such as bypass grafting or stenting since the long term patency is significantly limited by negative vessel remodeling.[1]

Normally, VSMCs are in a contractile state (i.e., quiescent / non-proliferating) and form direct contacts with ECs.[6] VSMCs express high levels of cytoskeleton stabilizing proteins, such as Smooth muscle α-actin (ACTA2), SM22α, SM MHC, Calponin1 (CNN1), Smoothelin and H-caldesmon. In addition, the contractile state is characterized by very low cell division rates and a very restricted migratory capacity of VSMCs. The typical response of VSMCs to any type of injury is a change of their phenotype from contractile to synthetic; they rearrange their cytoskeleton, proliferate and lose expression of cytoskeleton stabilizing genes such as *Cnn1* and *Acta2*.[7] The role of VSMC cytoskeleton stabilizing genes is highlighted in patients with mutations in the *Acta2* gene or its promoter, leading to a higher risk for coronary disease.[8, 9]

The VSMC phenotype switch has already been investigated in cell culture and is readily induced by growth factors such as PDGF, platelet-derived factors or inflammatory mediators such as TNF.[7, 10–12] Other mechanisms which are known to stimulate VSMCs in a way to trigger phenotype conversion are hypoxia and the resulting reactive oxygen species (ROS) generation.[13] However, simple cell culture approaches such as the culture of VSMCs do not accuaretly portray vascular injury as vital players of inflammation, platelets and endothelial cells are not part of the equation.[14]

*In vivo* experiments mainly have focused on long term outcomes such as restenosis and therefore do not elucidate the immediate response of blood vessels to injury. In addition, most models cause severe vascular damage such as constant pressure overload (venous bypass) or structural damage such as endothelial denudation (wire injury). In these models VSMCs are exposed to continuous stress over a period of days to weeks, which makes it difficult to analyze the initial vessel response and its resolution.[15, 16]

In this publication we have compared the responses of blood vessels subjected to bypass grafting or transient arterial ligation. These diverse surgical techniques yielded surprisingly comparable results within the first 24h after injury in terms of a transient inflammatory response and a persistent VSMC phenotype change. Extending the observation period to 28 days showed that temporary arterial ligation was sufficient to induce both, persistent changes in VSMC morpholgy and mRNA expression. Furthermore, mild IH occured, which was resistant to treatment. Most importantly, transient arterial ligation in a hypercholesteremic environment led to overt atherosclerotic lesion formation in the injured segments.

## Materials & methods

### Mice

8–10 week old, male C57BL/6J mice were housed for 2 weeks on a light/dark (12-hour/12-hour) cycle at 24°C and received food and water *ad libitum* before experimentation. All experiments were performed according to protocols approved by the Institutional Committee for Animal Research and Care at the Medical University of Vienna (BMWF-66.009/0266-WF/V/3b/2014).

13–18 week old, male ApoE knockout mice were housed for 2 weeks on a light/dark (12-hour/12-hour) cycle at 24°C and received food and water *ad libitum* before experimentation. 2 weeks prior to surgery the diet was changed to a western diet (E15723-34, ssniff Spezialdiäten GmbH, Germany) which was maintained until 4 weeks after surgery, when the experiment ended.

### Bypass grafting, carotid artery mobilization and transient ligation procedures

Heparin (6U) was injected subcutaneously 60min prior to surgery. Narcosis was initiated by intraperitoneally injection of 0,3mg/kg Medetomidine, 1mg/kg Midazolam, 0,03mg/kg Fentanyl and 10mg/kg Ketamine. After surgery, the narcosis was antagonized by subcutaneous injection of 1mg/kg Atipamezole and 0,1mg/kg Flumazenil. After surgery, Buprenorphine was injected subcutaneously for analgesic purpose. To maintain body temperature, all surgical procedures were performed on a heat plate (37°C). After surgery, mice were placed under a heat lamp to aid recovery.

Bypass grafting was performed as described previously.[16] Briefly, the carotid artery was mobilized, double-ligated, cut between the ligations and the ends were pulled through plastic cuffs, temporarily fixed in place via clamps, flipped over the cuffs and fixed in place. The inferior vena cava, excised from a donor mouse, was pulled over the cuffs and fixed in place. Following removal of the clamps, pulsatile flow was monitored and the wound sutured.

For the ligation procedure, the right common carotid artery was mobilized, care was taken to leave the adventitia in place and transiently ligated at the most upper and lower accessible end using removable slip knots made from 8–0 silk thread. Reperfusion was restored by thread removal after 20min or 60min as indicated. Sham animals were subjected to the same surgical mobilization procedure, but without ligation of the carotid artery.

For Sorafenib treatment, (CT-SR001, Chemietek, Indianapolis, IN, USA) Sorafenib was solubilized in Dimethyl sulfoxide (DMSO) and a 1μM Sorafenib solution (in 0.09% NaCl) was applied to the wound cavity during surgery. After ligation removal, a 20% w/v pluronic gel (P2443, Sigma-Aldrich, Darmstadt, Germany) containing 1μM Sorafenib/DMSO was placed around the artery, followed by wound closure. For Cytosporone-B treatment, the same procedure as for Sorafenib was applied with a Cytosporone-B concentration of 3μM in NaCl as well as 3μM in pluronic gel. For anti-TNF treatment, 100μg anti TNF antibody (MP6-XT3) or sham antibody (Rat IgG1 kappa Isotype Control, 16-4301-81, Thermo Fisher Scientific) was injected intraperitoneally 12h prior to surgery as well as 1h prior to surgery. For N-Acetylcysteine (NAC) treatment, 400mg/kg NAC/sham (saline) in a volume of 100μl was injected intravenously 5min before the 20min ligation period started. Animals were sacrificed at indicated times.

### Histology and immunofluorescence

Excised vascular segments were dissected to exclude regions where ligations were placed or anastomoses. For samples from ApoE knockout mice, the segment between the ligations, as well as the zone of bifurcation was analyzed. Vascular segments were fixed in 4% paraformaldehyde, embedded in an upright position in paraffin, cut sequentially into 4μm sections and subjected to hematoxylin and eosin staining (H&E) or immunofluorescence staining. The distance between sections ranged from 200μm to 300μm. For immunofluorescence, the following antibodies were used at indicated concentrations: anti-ACTA2 (Abcam, ab21027) (1:1000), anti-ICAM-1 antibody [YN1/1.7.4] (Abcam, ab119871) (1:200). Secondary antibodies were Alexa Fluor 546 (Invitrogen, A11056) and Alexa Fluor 647 (Jackson Immuno-research, 712-606-153), both utilized at a 1:200 dilution.

Quantification of nuclear sizes was performed by ImageJ on H&E sections. IH and atherosclerosis were analyzed on H&E sections.

### mRNA expression

Following excision of the venous bypass grafts, the anastomosis zones were removed and the grafts were subjected to RNA sequencing. Sequencing libraries were prepared by the Core Facility Genomics at the Medical University of Vienna using the SMARTer Ultra Low Input RNA for Illumina Sequencing in combination with the Low Input Library Prep Kit (Promega). Libraries were sequenced on the Illumina HiSeq 2000 platform in the 50 bp SR Modus. fastqc (http://www.bioinformatics.babraham.ac.uk/projects/fastqc/) (version 0.11.5) was used to check the quality of reads. Reads were mapped to mouse genome reference (mm9) using gsnap [17, 18] (version 2013-05-09) with the non-default parameter: max-mismatches (−m) 10. For Expression quantification and differential expression analysis counted reads were mapped to each gene (counts) using HTSeq-count[19] (version 0.5.4p3) with parameters:—stranded = no, -a 19 and a mouse GTF file downloaded from UCSC. Differential expression (DE) analysis was performed using DESeq2[20] (version 1.14.1) package in R. For pathway analysis, the genes tested for DE were connected with pathways from Reactome pathway Knowledgebase.[21] For each reactome pathway, which had 20 to 80 genes, a *pathway score* was calculated, as an average value of *log2fold* changes (logarithm with basis 2 of fold changes) of genes belonging to that pathway, normalized by standard deviation (sd) of these *log2fold* changes. Pathways were ranked based on their *pathway score* (from highest to lowest value). Significantly up-regulated pathways were defined as the top 10% pathways in the ranked pathway list. Similarly, significantly down-regulated pathways are the bottom 10% pathways in the ranked pathway list.

Using the MsigDB_XML_Browser-1.0_beta software[22] (msigdb_v6.1,), from the Curated and Gene Ontology (GO) collections all those gene sets related to the term “smooth muscle” were extracted and subjected to further analysis. Human gene symbols were converted to mouse gene symbols using an in-house bash script based on the Gnu Parallel tool[23] and the ortholog information (Ensembl Biomart version 87).

As p-value and log fold change (LogFC) is often used to evaluate significant results from differential expression analysis and the up/down-regulated genes are usually at the top/bottom of the ranked gene list, we use the signed -log_10_(p-value) to rank genes, where the sign is from LogFC, as previously described.[24]

To assess the enrichment of the smooth muscle related gene sets, in the different time points, GSEA Preranked tool was used.[22, 25] Only gene sets showing a significant change (FDR ≤ 0.05) in any assessed time point are displayed.

Heat maps were produced either using the R package ggplot2[26], or Morpheus, a matrix visualization and analysis software developed by the Broad Institute (https://software.broadinstitute.org/morpheus/). Results of the RNA sequencing analysis are available at the GEO database by utilizing the following link: https://www.ncbi.nlm.nih.gov/geo/query/acc.cgi?acc=GSE119549.

For rtPCR, Taqman assays on demand from Thermo-Fischer Scientific were used (*E-Selectin* [Mm01310197_m1], *Icam-1* [Mm00516023_m1], *Cnn1* [Mm00487032_m1], *Acta2* [Mm01546133_m1], *Interleukin1 beta* [Mm00434228_m1], *Interleukin6* [Mm00446190_m1], *Cxcl*1 [Mm04207460_m1], *S100a9* [Mm00656925_m1], *Cd45* [Mm01293577_m1], *Nr4a1* [Mm01300401_m1], *Pdgf β* [Mm00440677_m1], *Hif1α* [Mm00468869_m1] *B2m* [Mm00437762_m1]). Fold change was calculated using the 2^-ΔΔCT^ method as described previously.[27]

### Statistics

Statistical analysis was performed using GraphPad Prism version 5.03 for Windows, GraphPad Software, San Diego California USA. Respective statistical tests are shown in the legend of each figure. Data is presented as mean ± SD. A P-value <0,05 was considered as statistically significant (*) (<0,01 = **; <0,001 = ***).

## Results

### Bypass surgery and transient ligation, both induce transient inflammation and VSMC activation within 24h

To screen for immediate changes in a vessel subjected to bypass grafting, a vein graft model was used, which consistently induces intimal hyperplasia within 28 days.[16] mRNA sequencing analysis was performed on bypass grafts obtained 1, 6 and 24h after grafting to monitor the immediate reaction of the tissue to injury.

**Fig 1:**
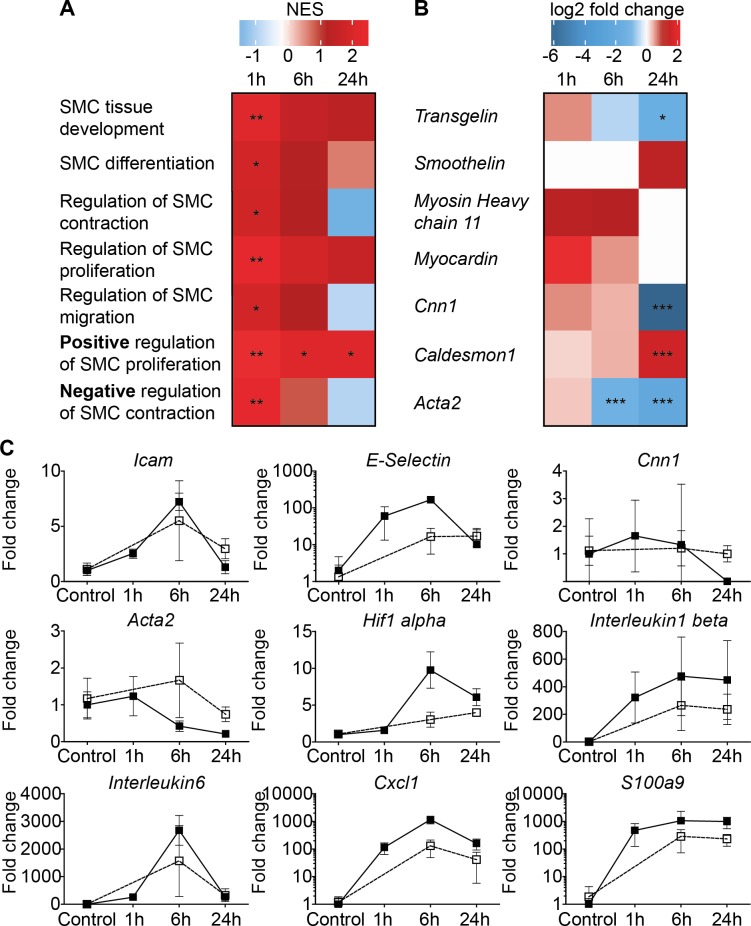
Comparison between bypass grafting and transient arterial ligation. RNA sequencing pathway analysis of venous bypass grafts 1, 6, and 24h after transplantation. Data are expressed as log2 fold change compared to normal vena cava. (A) Analysis of smooth muscle cell associated pathways in response to bypass grafting. (B) Regulation of mRNA expression of genes related to smooth muscle cell quiescence in response to bypass grafting. (C) mRNA expression of venous bypass grafts (filled squares) and transiently ligated carotid arteries (transparent squares; analyzed via qPCR) after indicated reperfusion times. Data are expressed as fold change compared to normal carotid arteries. For bypass grafts and transiently ligated carotid arteries n≥4.

Analysing all pathways associated with smooth muscle cell behaviour revealed significant changes (Fig 1A). Most importantly, the pathway responsible for positive regulation of SMC proliferation was uniformly upregulated as early as 1h post-surgery. In addition, an individual assessment of genes associated with the VSMC phenotype switch was performed (Fig 1B). *Transgelin*, *Cnn1* and *Acta2* showed a significant downregulation at 24h post-surgery, which indicates a phenotypic switch from contractile to synthetic.

Vein grafts are exposed to extracorporeal storage, surgical trauma, reperfusion injury and persistent pressure overload.[14] To reduce variables, we compared effects of vein grafting with those induced by transient carotid artery ligation, which exposes the vessel to surgical trauma, ischemia and reperfusion injury. Importantly, changes in gene expression within the first 24h post-surgery were comparable to those observed after vein grafting (Fig 1C).

### Transient Carotid artery ligation induces a permanent loss of VSMC quiescence gene expression

**Fig 2:**
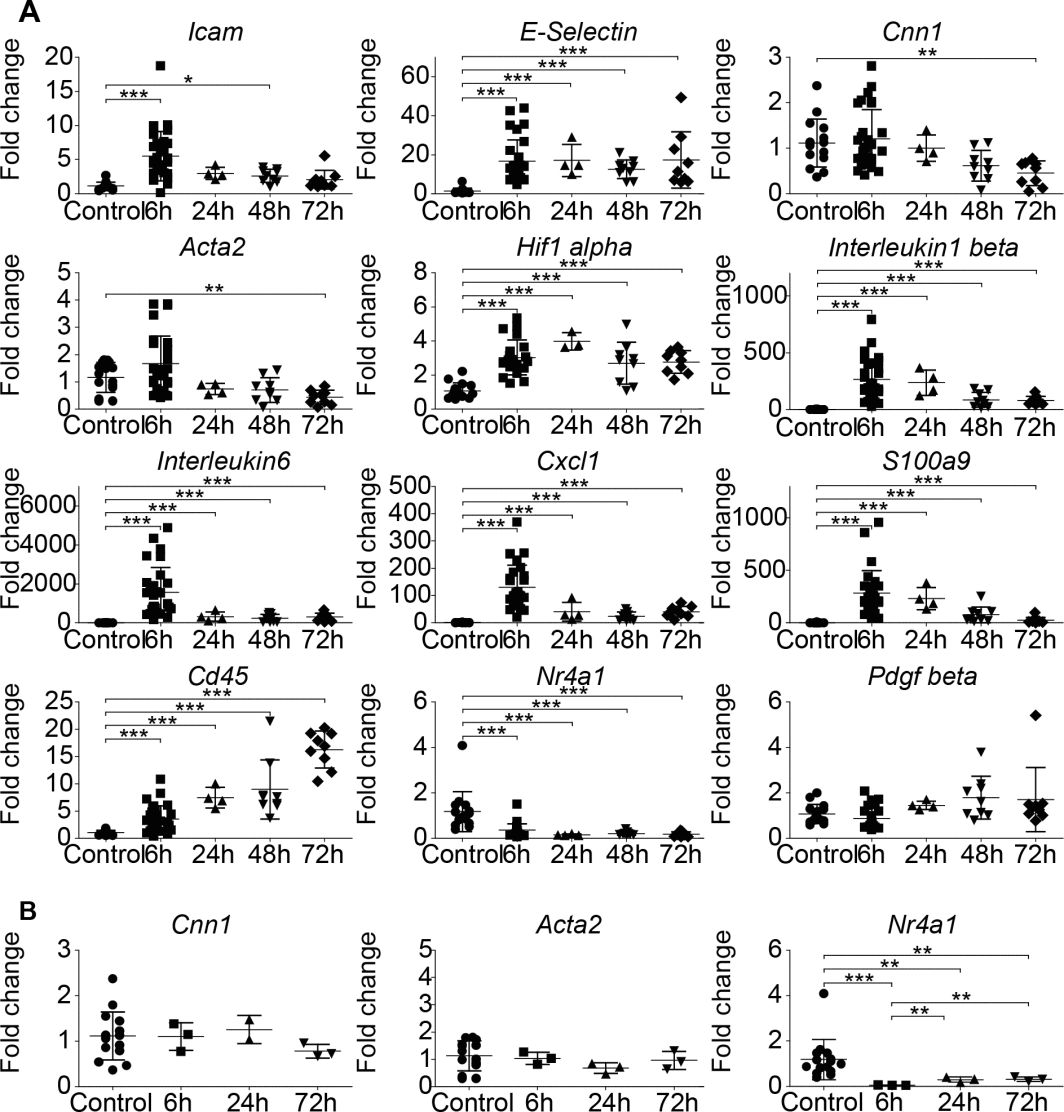
Kinetics of the vascular response following transient ligation. (A) mRNA expression of right carotid arteries in response to 20min temporary ischemia induced by transient ligation. (B) Analysis of mRNA expression pattern in response to surgical mobilization of the right carotid artery without ligation. Statistical evaluation was performed by using a one-way ANOVA with Tukey’s multiple comparison post hoc test; n = each symbol represents one mouse.

Extending the follow-up period after transient carotid artery ligation revealed a persistent VSMC phenotype switch, as indicated by a significant loss of *Acta2* and *Cnn1* mRNA expression after 72h, compared to untreated carotid arteries (Fig 2A). mRNA expression of inflammatory genes peaked after 6h, followed by a steep decline close to baseline levels. The amount of *Cd45* mRNA expression, a leucocyte marker, steadily increased. As expected, ligation increased mRNA expression of the transcription factor *Hif1α*, which is known to trigger expression of a multitude of growth factors.[28] Interestingly, expression of *Nr4a1* mRNA, an important gene regulating the homeostasis in the vessel wall was reduced to almost zero.[29, 30]

**Fig 3:**
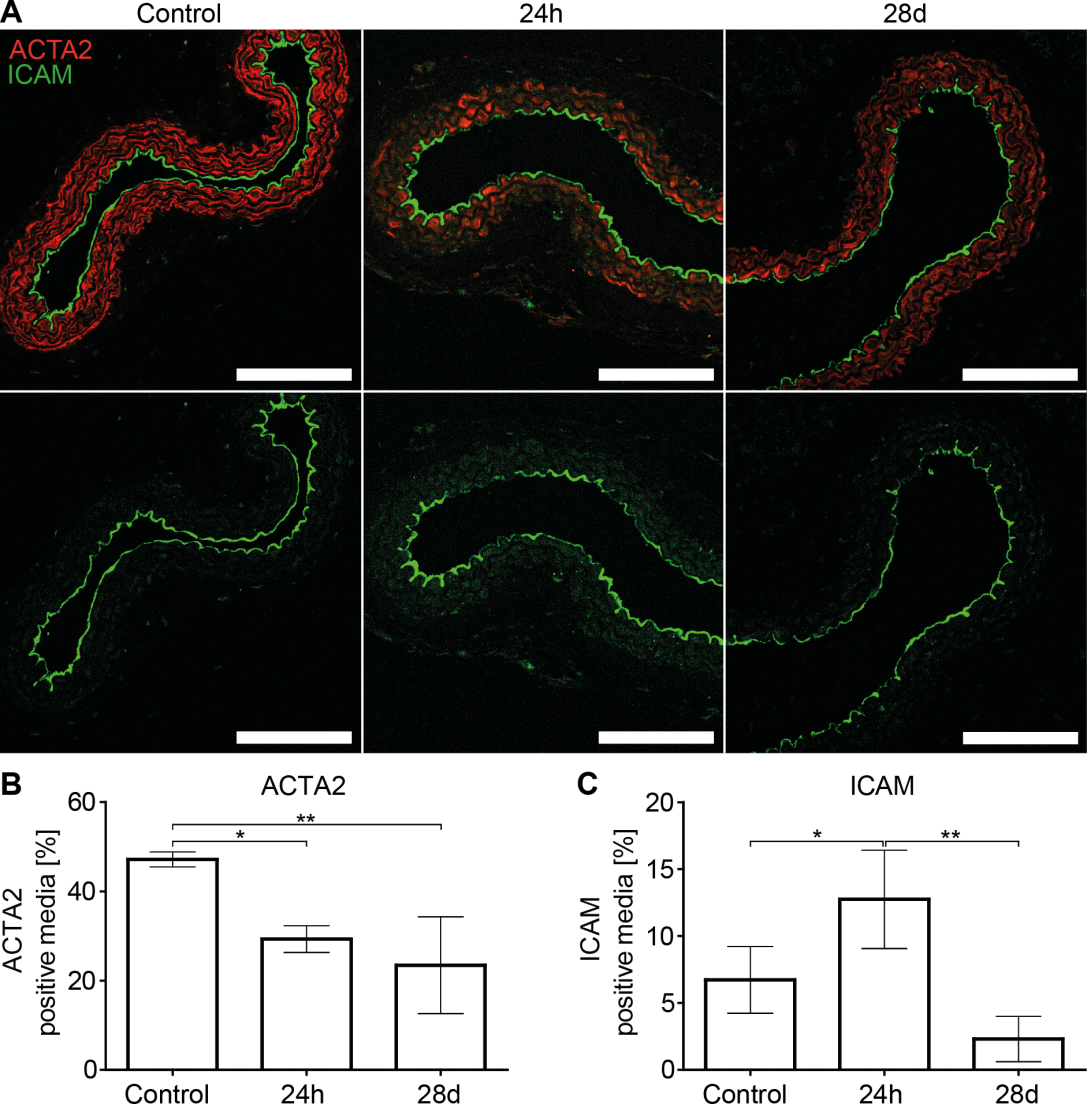
Transient ligation induces a persistent loss of ACTA2 protein. (A) representative ICAM-1 and ACTA2 stainings of transiently ligated carotid arteries analyzed after 24h (20 min transient ligation) or 28 days (1h transient ligation). Scale Bar equals 100μm. (B) Quantification of ACTA2 positive media (n = 3–4 per time point) (C) Quantification of ICAM-1 positive media (n = 3–4 per time point). Statistical analysis was performed using a one-way analysis of variance with Tukey’s multiple comparison post hoc test.

As a control, carotid arteries were exposed to surgical trauma only (vessel mobilized without ligation). This procedure did not alter the expression of VSMC quiescence gene mRNAs, but suppressed *Nr4a1* mRNA expression (Fig 2B), excluding *Nr4a1* as a driver of *Acta2* or *Cnn1* expression. To investigate if an altered mRNA expression is followed by an altered protein expression, immunofluorescence stainings of temporarily clamped carotid arteries were performed. This confirmed a transient increase in ICAM-1 protein expression and a loss of ACTA2 protein expression by VSMCs (Fig 3A–3C).

**Fig 4:**
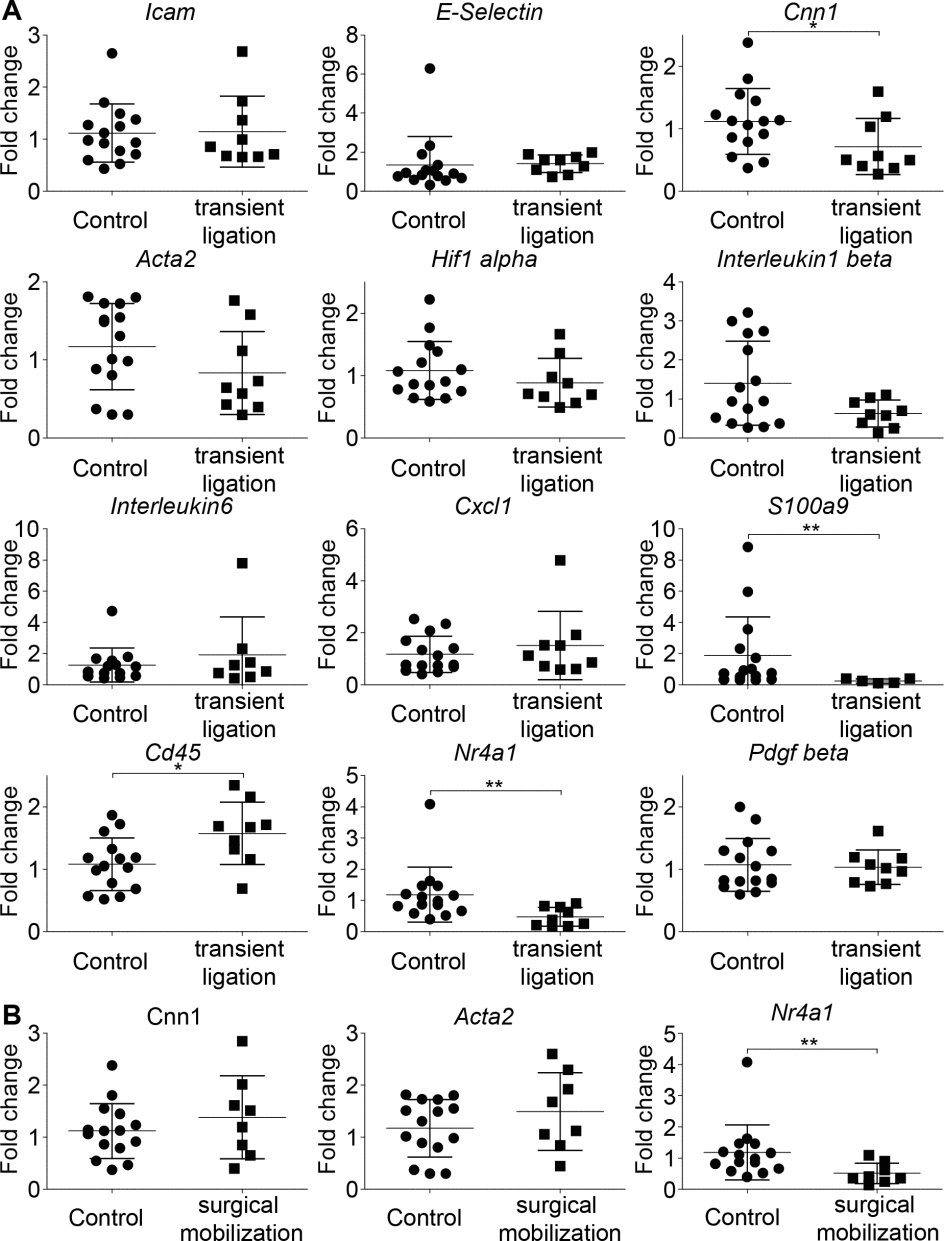
Long term response of arteries subjected to transient clamping. (A) Analysis of mRNA expression patterns of carotid arteries subjected to 1h transient ligation followed by 28 days of reperfusion. (B) Analysis of mRNA expression patterns of carotid arteries subjected to surgical mobilization only, followed by 28 days of reperfusion. Statistical analysis was performed using a two-tailed unpaired t-test. n = each symbol represents one mouse.

For the 28 day follow-up experiments carotid arteries were clamped for 1h (an ischemia time frequently encountered during CABG surgery[31]). mRNA expression profiles are displayed in Fig 4A. 28 days post-surgery, mRNA expression of inflammatory genes was back to baseline, whereas *Cnn1* mRNA was still significantly downregulated compared to untreated control arteries. In case of *Acta2* mRNA, the downregulation did not reach the threshold of significance, however, semi quantitative evaluation by immunofluorescence demonstrated a significant loss of ACTA2 protein expression after 28 days (Fig 3A and 3B). *Cd45* mRNA remained significantly upregulated, indicating an accumulation of immune cells. *Nr4a1* mRNA, remained significantly downregulated.

In controls subjected to surgical mobilization of the carotid artery without ligation, no effects on VSMC quiescence gene mRNA expression were observed. Unexpectedly, expression of *Nr4a1* mRNA remained significantly downregulated, indicating that the loss in *Nr4a1* mRNA expression is not directly responsible for mRNA expression levels of *Acta2* and *Cnn1* (Fig 4B).

**Fig 5:**
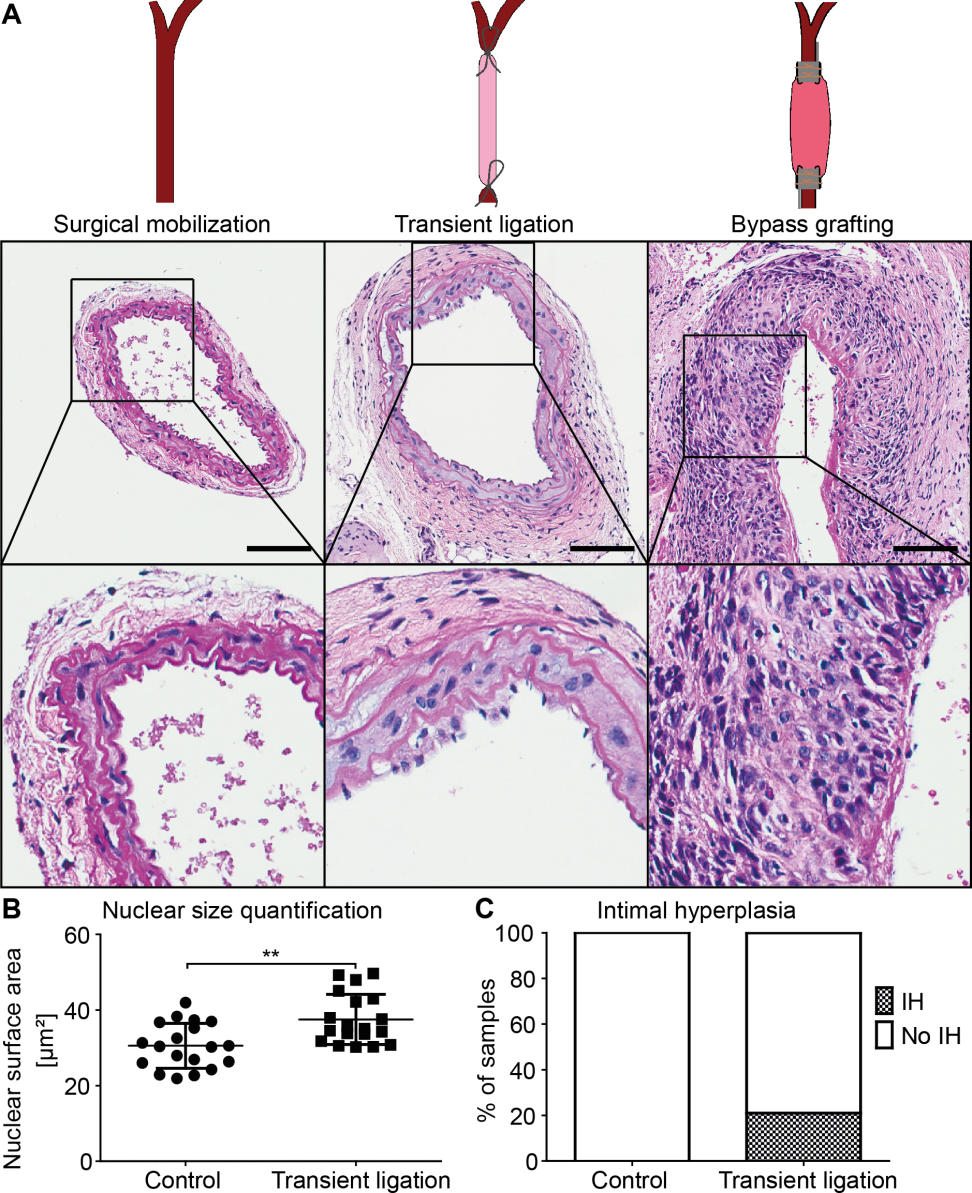
Morphological analysis of transiently ligated arteries. (A) Examples of carotid arteries subjected to surgical mobilization, to 1h of transient ligation or to bypass grafting (inferior vena cava grafted into the carotid circulation). 28 days after surgery, blood vessels were removed and subjected to H&E staining. Scale bars equal 100**μ**m. (B) Quantification of nuclear area of VSMCs within the media of carotid arteries subjected to 1h transient ligation followed by 28 days of reperfusion, compared to untreated contralateral control arteries. Statistical analysis was performed using a two-tailed unpaired t-test. (C) Evaluation of the presence of an enlarged intimal layer of carotid arteries subjected to 1h transient ligation followed by reperfusion for 28 days. As controls, the untreated contralateral arteries were utilized; n = 19.

To assess morphologic consequences of the monitored VSMC phenotype switch, histologic analysis was conducted to assess vessel remodeling (Fig 5). In 20% of cases, the clamped artery showed an enlarged intimal layer. In addition, the nuclear area of medial VSMCs was significantly enlarged compared to the contralateral carotid artery. We viewed this as an indirect sign of polyploidy, a repeatedly observed phenomenon after vascular injury.[32] As a control we analyzed arteries subjected to surgical mobilization without ligation, which showed no morphological alterations. For comparison, a segment of an arterial bypass graft showing massive IH is also displayed.

### Transient vascular ligation is sufficient to precipitate atherosclerosis in hypercholesteraemic mice

Most patients requiring vascular interventions such as angioplasty or bypass grafting have elevated blood cholesterol levels.[33] We therefore analyzed the effects of vascular ligation in ApoE-deficient mice which were fed a Western diet 2 weeks before ligation until the end of experiment, 4 weeks after surgery.

**Fig 6:**
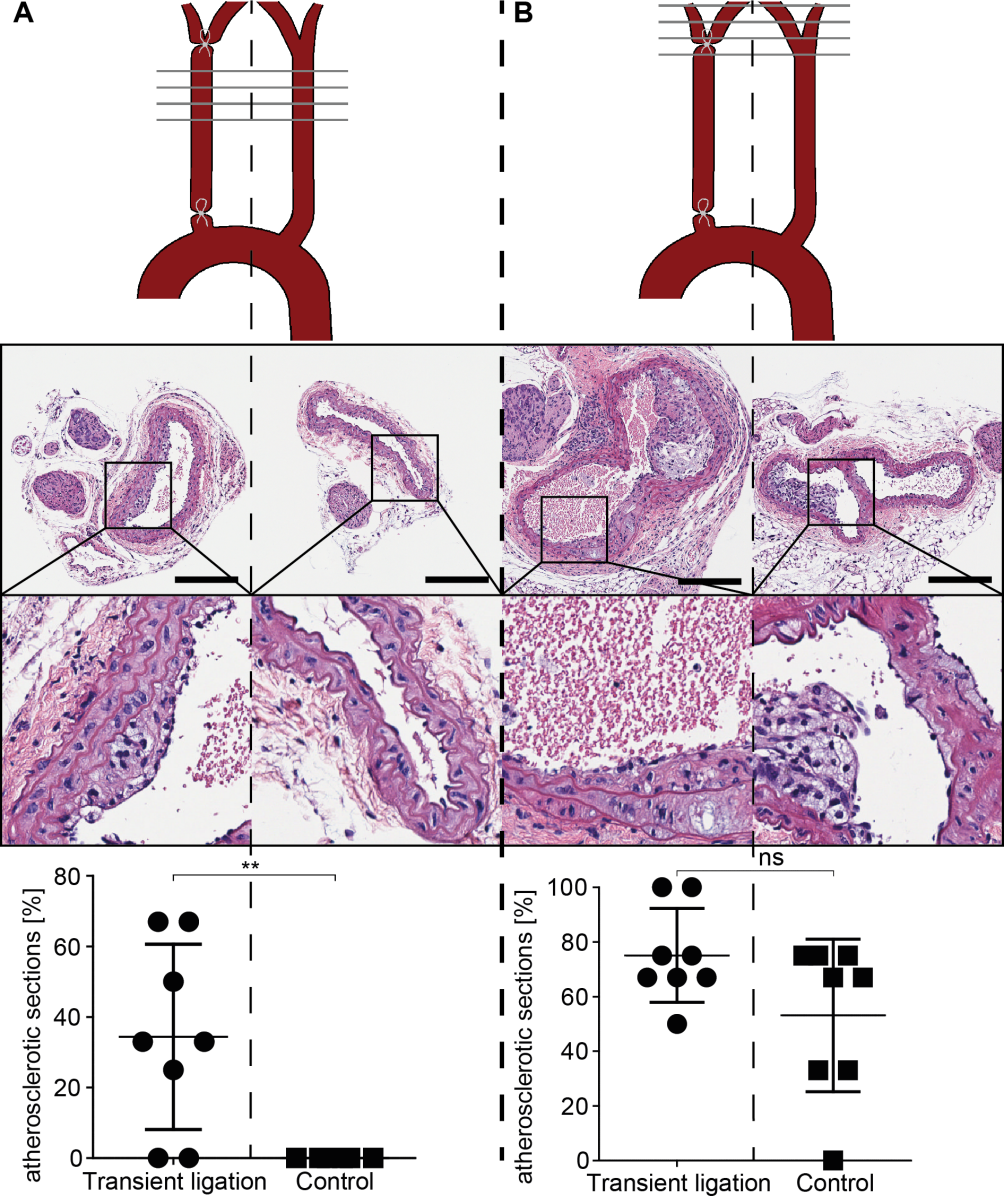
Transient ligation results in atherosclerosis in ApoE deficient mice. Effect of 1h transient carotid artery ligation on carotid arteries of ApoE deficient mice (fed a western diet), assessed after 28 days of reperfusion. Scale bar equals 200 **μ**m. (A) vascular segment with laminar flow; (B) vascular segment including the area of bifurcation. Per vascular segment, the area below bifurcation (A) and the area of bifurcation (B), 3–4 H&E sections were analyzed as indicated by the horizontal grey lines in the schematic drawing. n = 8, statistical analysis was performed using a two tailed paired t-test.

Atherosclerotic plaques were detected at predilection sites such as the carotid artery bifurcation in the left and right carotids. In contrast, while the left carotids (control side) remained free of lesions at regions of laminar flow, in right carotids (transiently ligated areas) atherosclerosis was evident (Fig 6, A gallery of all mice is displayed in S1 Fig).

### Pharmacological interventions do not prevent VSMC phenotype changes

**Fig 7:**
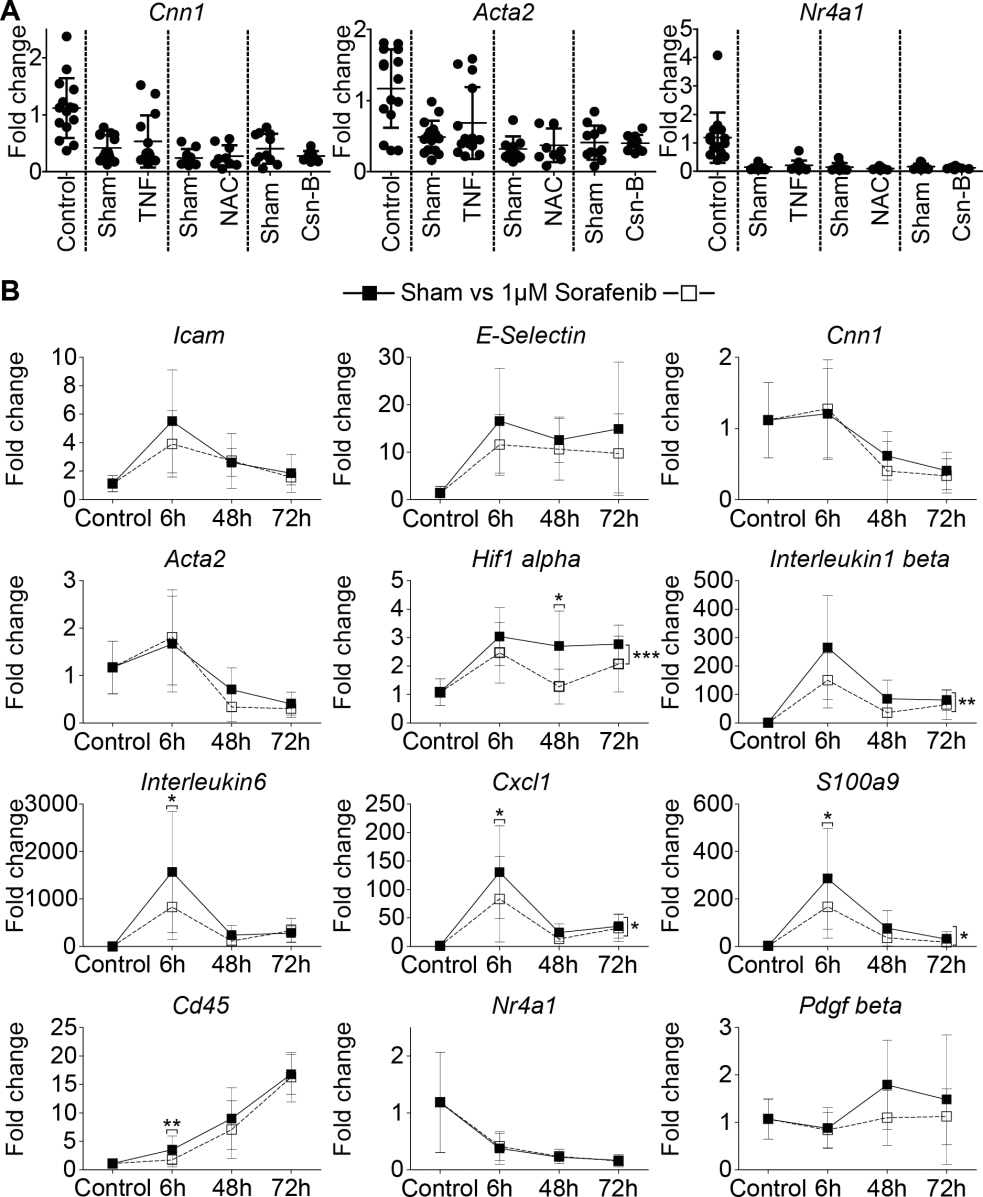
Pharmacologic interventions do not prevent the VSMC phenotype switch. (A) mRNA expression of right carotid arteries subjected to 20min transient ligation and 72h reperfusion. Mice were either treated with TNF scavenging antibody/sham antibody, N-Acetylcysteine/Saline or Cytosporone B/sham gel. Statistical evaluation was performed by using a one-way ANOVA with Tukey’s multiple comparison post hoc test. n≥9. (B) mRNA expression of right carotid arteries subjected to 20min transient ligation followed by reperfusion periods as indicated. Arteries were either exposed to 1**μ**M Sorafenib or sham (DMSO) treatment. Statistical evaluation was performed by using a two way ANOVA with Bonferroni’s post hoc test. n≥6.

Based on the finding that transient ligation but not surgical mobilization alone induces a persistent VSMC phenotype change, we concluded that hypoxia followed by reperfusion is the most important driver of persistent vascular remodelling. To analyze if pro-inflammatory cytokines trigger the VSMC phenotype switch, we treated mice with anti-TNF antibodies. Unexpectedly, this treatment did not prevent the ligation-induced loss of *Acta2* or *Cnn1* mRNA expression (Fig 7A).To assess the possibility that ROS induced the VSMC phenotype change we used N-Acetylcysteine (NAC) which is well known for its cytoprotective properties with regards to ROS-induced cell damage.[34] As displayed in Fig 7A, NAC had no effect on VSMC quiescence gene mRNA, or on *Nr4a1* mRNA expression. NR4A1 is an important transcription factor in maintaining a functional blood vessel.[29, 30, 35] However, an NR4A1 agonist, Cytosporone-B[36], had no effect on VSMC quiescence gene mRNA expression (Fig 7A). Finally, to test the importance of growth factor signalling, a multi tyrosine kinase inhibitor was employed. Sorafenib was originally developed as an anti-cancer drug aiming at growth suppression by inhibiting a multitude of different kinases, among which are VEGFR, PDGFR and Raf family kinases.[37] As shown in Fig 7B, 1μM Sorafenib significantly downregulated mRNA expression of *Il-1* and *6*, *Cxcl1*, *S100a9* and *Hif1α*, but did not prevent the downregulation of *Acta2*, *Cnn1* or *Nr4a1* mRNA, and therefore did not prevent the VSMC phenotype switch.

## Discussion

The VSMC phenotype change from contractile to synthetic is a typical response of the vascular wall to any type of vascular injury. Synthetic VSMCs migrate and proliferate to promote repair. Under normal conditions they return back to a non-proliferative, contractile phenotype. However, this plasticity renders VSMCs susceptible to pathological growth resulting in IH.[7] A prolonged or persistent VSMC phenotype switch contributes to initiation and progression of vascular pathologies such as atherosclerosis, post-angioplasty re-stenosis, and pulmonary arteriole hypertension.[38–40] Even in completely different settings VSMC IH is causative for disease as seen, e.g., in the development of cerebral vasospasm after subarachnoid hemorrhage or blast traumatic brain injury.[41–43] Recent observations also link VSMC phenotype changes to tumor niche formation resulting in an elevated metastatic potential in cancers. Tumor cells shed mediators inducing a VSMC phenotype conversion which facilitates metastasis into these areas.[44] Because of this variety of conditions interrelated with abnormal plasticity of VSMCs *in vivo* models are required to understand (i) factors inducing the phenotype switch, (ii) factors maintaining this pathological condition and (iii) how to therapeutically revert the VSMC phenotype back to quiescence.

Based on the diversity of events that may lead to IH we hypothesized that the type of initial trauma is responsible for the outcome. We first have analyzed molecular changes occurring within the first 24h after vessel injury in a venous bypass model, which is prone to induce IH.[14, 16] Surprisingly, the inflammatory response was transient peaking after 6h, whereas the loss in the expression of VSMC differentiation genes persisted. Since this procedure exposes the grafted vessel to surgical injury, *ex vivo* graft storage, endothelial denudation, hypoxia/reperfusion, pressure and flow changes, we next used a model with as little confounding factors as possible.[14] A carotid artery ligation model which inflicts only very limited vascular damage, leaving the adventitia in place and not causing endothelial denudation. The transiently ligated vascular segment is mainly exposed to hypoxia/reperfusion. Surprisingly, RNA expression changes within the first 24h were very similar to those seen in vein grafts. Extended time kinetics in the ligation model revealed a persistent phenotype change throughout 28 days, which was not seen in controls, where the carotid artery was subjected to surgical exposure without ligation. This points to the conclusion that hypoxia/reperfusion modulates VSMCs to switch to a synthetic phenotype. Moreover, this type of injury was sufficient to induce subtle morphologic changes within the transiently ligated segment of the carotid arteries, namely significant nuclear size enlargement of medial VSMCs. In addition 20% of the samples displayed mild intimal hyperplasia/cell accumulations. Nuclear size enlargement may be an indirect sign for polyploidy since it has been proposed that polyploidization is a normal, controlled, protective mechanism against oxidative stress.[45, 46] Specifically, the exposure of cells to mitotic stimuli in the presence of oxidative stress can result in polyploidy.[32, 47] Oxidative stress and ROS are well documented in situations of ischemia/reperfusion.[48] Together with the development of IH in 20% of cases and the persistent downregulation of cytoskeleton stabilizing genes after 28days, this clearly demonstrates that a short-term trigger, such as hypoxia/reperfusion, alone is sufficient to cause a long lasting VSMC damage despite a fast resolution of the inflammatory reaction.

To test the physiological relevance of this persistent VSMC damage, ApoE knockout mice were employed. We are aware that the ApoE knockout mouse has several limitations compared to humans. Wild type mice have a low cholesterol level with high-density lipoprotein being the predominating component and only low amounts of pro atherogenic components such as low-density lipoprotein or very-low-density lipoprotein. Therefore, the mouse is resistant to atherosclerosis, especially since this favorable cholesterol ratio prevails even under high fat diet. Humans show higher cholesterol levels and low-density lipoprotein is the major constituent, resulting in a higher risk for atherosclerosis. In comparison, ApoE knockout mice develop exceedingly high hypercholesteremia under western diet (~1800mg/dl), mainly driven by an increase in very-low-density lipoprotein.[49, 50] In addition, the ApoE protein does not only participate in lipoprotein metabolism, but also influences inflammation and anti-oxidant activities, which are missing in the knockout mouse.[51] However, the absence of a multifactorial scenario as given in this genetic mouse model allows describing effects of a stress signal (ischemia/reperfusion) with or without hypercholesterinemia.

These mice display a significantly elevated blood cholesterol level which can be further increased under a western diet.[49] Most patients subjected to revascularization techniques such as angioplasty or bypass grafting suffer from elevated cholesterol levels, diabetes and an elevated blood pressure.[33] ApoE knockout mice developed atherosclerotic lesions at the carotid artery bifurcation in the transiently ligated as well as in the contralateral control arteries. Since the carotid artery bifurcation is prone to formation of atherosclerotic lesions due to flow changes, this result was expected. The vascular segment below the carotid bifurcation is usually not prone to atherosclerotic plaque formation.[52, 53] Indeed, contralateral control arteries, which were not subjected to temporary ligation were free of lesions. In contrast, arteries transiently ligated for 1h and explanted 4 weeks later showed atherosclerosis. This indicates that persistent VSMC activation due to transient ligation primes the blood vessel for the formation of atherosclerotic lesions in a hypercholesteremic environment.

Ischemia/reperfusion injury is characterized by hypoxia, triggering expression of hypoxia inducible genes such as HIF1α and formation of ROS, triggering inflammation.[54] HIF1α acts as a transcription factor for various growth factors, such as PDGF which is known to induce VSMC phenotype conversion.[7, 28] To elucidate mechanisms that initiate a synthetic VSMC phenotype, we employed several treatments. A TNF scavenging antibody did not prevent phenotype conversion. To counteract ROS, N-Acetylcysteine was used, which also had no effect on phenotype switching. The significant and persistent downregulation of *Nr4a1* mRNA in clamped arteries prompted us to use an NR4A1 agonist, Cytosporone B[36], since NR4A1 downregulation promotes a VSMC phenotype switch and NR4A1 knock out mice show an increased proliferative VSMC behavior in response to pro-proliferative signals.[35, 55] However, Cytosporone B treatment did not affect the VSMC phenotype switch. The role of NR4A1 in initiating the phenotype switch is also questioned by our control experiment, subjecting the carotid artery to surgical dissection without ligation. In this model *Nr4a1* mRNA was also downregulated but the VSMC genes *Cnn1* and *Acta2* were not affected. Finally, we used a compound originally developed as an anti-cancer treatment which inhibits a wide variety of kinases, mainly growth factor related signaling cascades such as VEGFR, PDGFR and Raf family kinases.[37] Specifically PDGFR signaling is thought to play an important role in the VSMC phenotype switch, but Sorafenib did not prevent downregulation of *Cnn1* and *Acta2*, albeit it significantly downregulated *Il-1*, *Il-6*, *Cxcl1*, *S100a9* and *Hif1α* mRNA.[7] These results point to the conclusion that single triggers such as growth factors, TNF, ROS or the loss of NR4A1 can be ruled out as a cause of persistent phenotype conversion of VSMCs, albeit, in cell culture, each of these triggers induces a loss in expression of VSMC differentiation genes.[7, 35]

## Conclusion

The results of our simple and reproducible transient ligation model are unique in several aspects. The carotid artery is subjected to hypoxia/reperfusion without affecting the vascular architecture, the adventitia is left in place and there is no endothelial denudation. Nevertheless, this procedure induces a permanent VSMC phenotype change towards a secretory phenotype. This implies that (i) reperfusion injury occurs not only in large organs with a complex architecture, but even in such small “simply built” structures as the carotid arteries; (ii) efforts to prevent IH should not only aim to suppress inflammation but also need novel strategies to prevent the VSMC phenotype change. (iii) Sorafenib should be considered as a treatment option in surgical procedures, where the aim is to suppress inflammation since it can be locally employed at concentrations which are far lower than required for cancer treatment; (iv) developing strategies to prevent the VSMC phenotype change may also serve as supplementary therapy in treatment of certain cancer types which utilize activated VSMCs to generate a favorable metastatic niche. (v) our results demonstrate that any type of transient vascular ligation/clamping for surgical reasons causes vascular alterations. In situations of a perturbed lipid metabolism this bears the risk to precipitate atherosclerosis.

## Supporting information

S1 Fig Gallery of transiently ligated and contralateral control carotid arteries of ApoE knock out mice fed a western diet. H&E stainings; scale bar equals 200μm.
(TIF)

## Abbreviations

ACTA2: Smooth muscle α-actin
ApoE: Apolipoprotein E
CNN1: Calponin
DMSO: Dimethyl sulfoxide
EC: Endothelial Cell
HIF: Hypoxia Inducible Factor
H&E: Hematoxylin and eosin stain
IH: Intimal Hyperplasia
ROS: Reactive Oxygen Species
VSMC: Vascular Smooth Muscle Cell
