# Supplementary figures and images for: Hypoxia/reperfusion predisposes to atherosclerosis
Source: PLoS One. 2018 Oct 5;13(10):e0205067. doi: 10.1371/journal.pone.0205067 (PMC6173417; doi:10.1371/journal.pone.0205067)

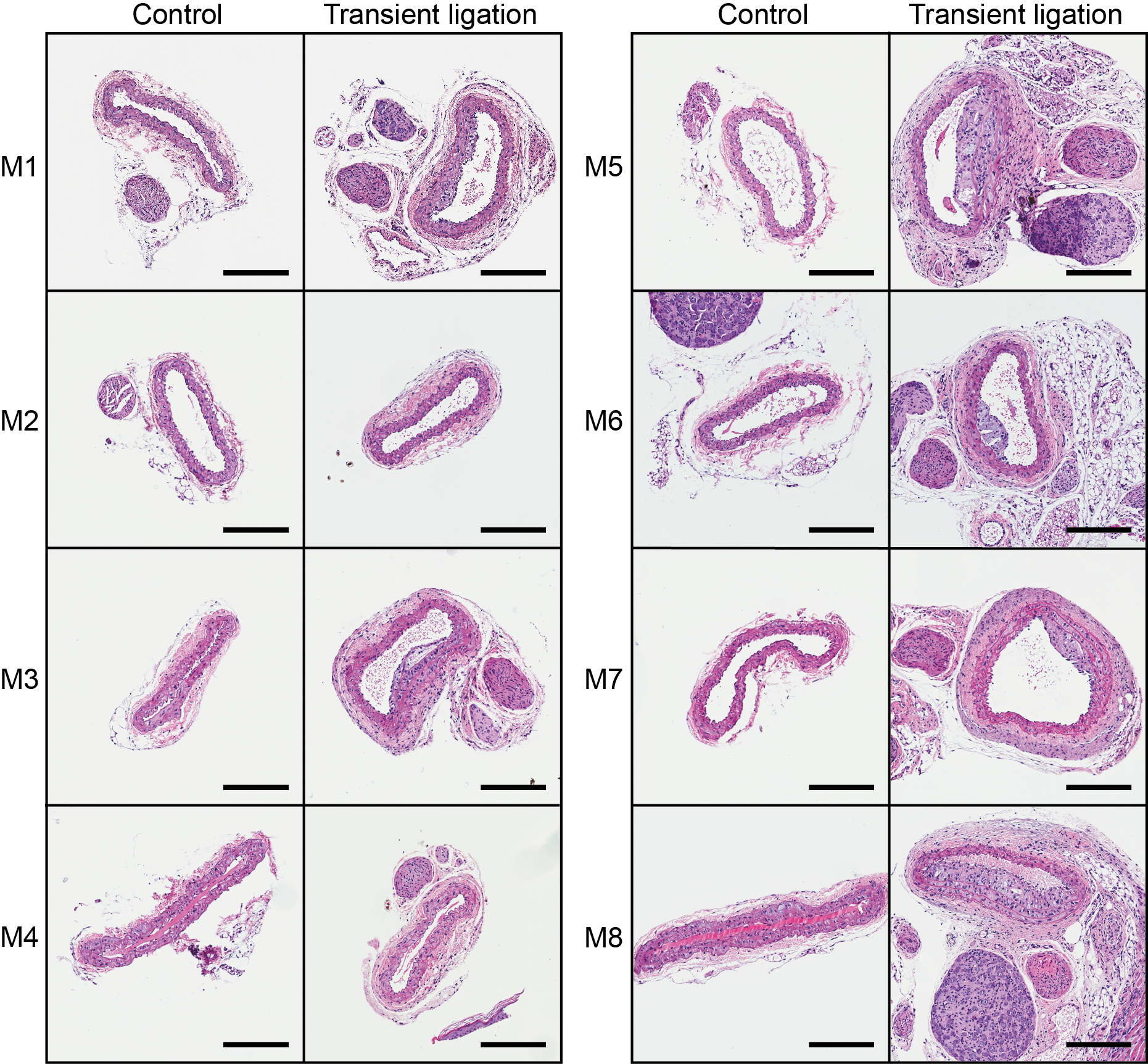

Supplement: S1 Fig — H&E stainings; scale bar equals 200μm. (TIF) [file pone.0205067.s001.tif]
